# Removal of the product from the culture medium strongly enhances free fatty acid production by genetically engineered *Synechococcus elongatus*

**DOI:** 10.1186/s13068-017-0831-z

**Published:** 2017-05-31

**Authors:** Akihiro Kato, Nobuyuki Takatani, Kazutaka Ikeda, Shin-ichi Maeda, Tatsuo Omata

**Affiliations:** 1 Graduate School of Bioagricultural Sciences, Nagoya University, Nagoya, 464-8601 Japan; 2 Laboratory for Metabolomics, RIKEN Center for Integrative Medical Sciences, Yokohama, 230-0045 Japan; 3 Japan Science and Technology Agency, CREST, Tokyo, Japan; 4 Laboratory of Molecular Plant Physiology, Graduate School of Bioagricultural Sciences, Nagoya University, Furo, Chikusa, Nagoya, 464-8601 Japan

**Keywords:** Cyanobacteria, Biofuel production, Free fatty acids, Two-phase culture system

## Abstract

**Background:**

Cyanobacterial mutants engineered for production of free fatty acids (FFAs) secrete the products to the medium and hence are thought to be useful for biofuel production. The dAS1T mutant constructed from *Synechococcus elongatus* PCC 7942 has indeed a large capacity of FFA production, which is comparable to that of triacylglycerol production in green algae, but the yield of secreted FFAs is low because the cells accumulate most of the FFAs intracellularly and eventually die of their toxicity. To increase the FFA productivity, enhancement of FFA secretion is required.

**Results:**

Growth of dAS1T cells but not WT cells was inhibited in a liquid medium supplemented with 0.13 g L^−1^ of palmitic acid. This suggested that when FFA accumulates in the medium, it would inhibit the release of FFA from the cell, leading to FFA accumulation in the cell to a toxic level. To remove FFAs from the medium during cultivation, an aqueous-organic two-phase culture system was developed. When the dAS1T culture was overlaid with isopropyl myristate (IM), the final cell density, cellular chlorophyll content, and the photosynthetic yield of PSII were greatly improved. The total amount of extracellular FFA was more than three times larger than that in the control culture grown without IM, with most of the secreted FFAs being recovered in the IM layer. The cellular FFA content was decreased by more than 85% by the presence of the IM layer. Thus, the two-phase culture system effectively facilitated FFA secretion out of the cell. An average FFA excretion rate of 1.5 mg L^−1^ h^−1^ was attained in the 432 h of cultivation, with a total amount of excreted FFA being 0.64 g L^−1^ of culture. These figures were more than three times higher than those reported previously for the cyanobacteria-based FFA production systems.

**Conclusions:**

Removal of FFA from the culture medium is important for improving the productivity of the FFA production system using cyanobacteria. Further increase in productivity would require an increase in both the rates of FFA production in the cell and active FFA export across the plasma membrane.

**Electronic supplementary material:**

The online version of this article (doi:10.1186/s13068-017-0831-z) contains supplementary material, which is available to authorized users.

## Background

Photosynthetic microorganisms including cyanobacteria and eukaryotic algae appear to be promising hosts for sustainable biofuel production because they have a high capacity for oxygenic photosynthesis [1, 2]. Actually, some research efforts have reported that their oil yields per unit area are more than three times higher than those of land plants including corn and oil palm [3, 4]. However, commercial success of algae-based biofuel production has not been achieved yet, mainly because of its high processing cost. Although green algae can accumulate triacylglycerol (TAG) in the cells to a level corresponding to 50% of cell dry weight [5], the high costs in recovery of the cells from the growth medium and extraction of lipid from the cells hamper the commercialization of the TAG-based biofuel production [6, 7].

Unlike TAG production using green algae, production of free fatty acids (FFA) using genetically engineered cyanobacteria results in secretion of the product into the medium [8–10]. If the rate of FFA secretion is raised to a level comparable to the TAG production rate of algal cultures, the cyanobacterial system would be advantageous over the algal production system as the cost-intensive steps in biofuel extraction can be omitted. Construction of FFA-producing cyanobacterial mutants involves the following two gene manipulations. Firstly, the *aas* gene coding for acyl-ACP synthetase, which activates FFAs via esterification to acyl carrier protein (ACP), is inactivated to prevent the recycling of the FFAs released from membrane lipids [11, 12]. Secondly, a gene encoding a truncated form of the thioesterase from *Escherichia coli* (*‘tesA*) [13] is introduced to cleave the thioester bond of acyl-ACP [8]. Among the three model cyanobacterial species used for construction of FFA-producing strains, i.e., *Synechocystis* sp. PCC 6803, *Synechococcus elongatus* PCC 7942, and *Synechococcus* sp. PCC 7002, *Synechocystis* sp. PCC 6803 has been the most successful [8–10]. In this strain, FFA productivity was successfully increased by the additional gene manipulations aimed at weakening of the peptidoglycan layer, inactivation of the PHB biosynthesis pathway, overexpression of acetyl-CoA carboxylase, and expression of thioesterases from plants and bacteria. The resultant SD277 mutant secreted FFAs at an average rate of 0.44 mg L^−1^ h^−1^ for 450 h [8]. However, this figure was still much lower than the rate of TAG production attained by cultures of eukaryotic algae, e.g., *Chlorella vulgaris* and *Nannochloropsis gaditana*, ranging from 2 to 3 mg L^−1^ h^−1^ [14, 15].

Although all the FFA-producing strains constructed from *S. elongatus* PCC 7942 were less active in FFA secretion than the SD277 mutant of *Synechocystis* sp. PCC 6803 [10, 16], Kato et al. found that the dAS1T strain, constructed from *S. elongatus* PCC 7942 simply by inactivation of *aas* and introduction of *‘tesA*, has a much larger capacity to produce fatty acids; it turned out that unlike the SD277 strain that secrete ~90% of FFA out of the cell [8], dAS1T retains most of FFA in the cell [16]. Cultures of dAS1T were thus shown to produce FFAs at a rate of 1.9 mg L^−1^ h^−1^, while secreting FFA at a rate of 0.35 mg L^−1^ h^−1^ [16]. This resulted in intracellular accumulation of FFA to a toxic level, causing cell death in 240 h of cultivation. The total amount of secreted FFA was also less than half of that attained by SD277. Impairment of synthesis of the hydrophilic O-antigen layer on the cell surface partially rescued the early-death phenotype of dAS1T by facilitating FFA release from the cells, suggesting that enhancement of FFA secretion is important for the construction of an efficient and robust FFA production system using *S. elongatus* [16].

In this study, we developed an aqueous-organic two-phase culture system to remove FFAs from the medium during cultivation of dAS1T. The rate of FFA secretion and the total amount of secreted FFA in the two-phase system were more than three times higher than those reported for the SD277 strain, verifying the importance of removal of FFAs from the culture medium in the enhancement of FFA production. Effects of the two-phase culture system on growth and FFA productivity of the dAS1T strain are described and the strategy for further improvement of FFA production is discussed.

## Results

### FFA accumulation in the medium inhibits growth of dAS1T

**Fig. 1:**
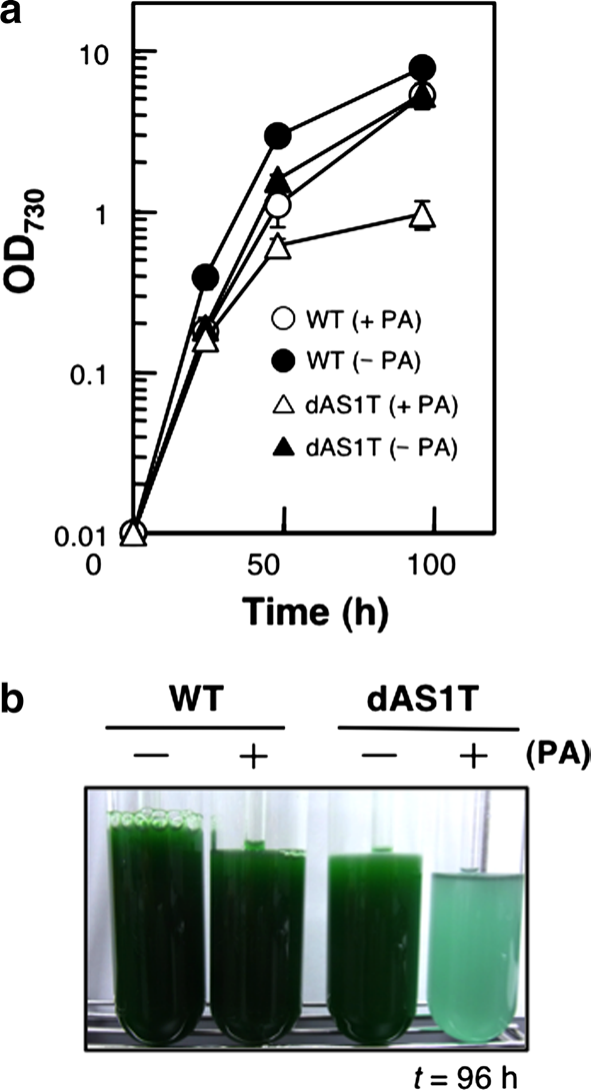
Effects of exogenously added palmitic acid on the growth of the WT and dAS1T cells. **a** Growth curves of WT (*circles*) and dAS1T (*triangles*). Cells were grown either with (+, *open symbols*) or without (−, *filled symbols*) 0.13 g L^−1^ of palmitic acid (PA). Data shown are the mean ± SE from biological triplicates. **b** Appearance of the cultures at *t* = 96 h, showing the growth defect of dAS1T grown in the medium supplemented with PA

Figure 1 shows the effects of exogenously added 500 µM (0.13 g L^−1^) of palmitic acid (PA) on growth of the wild-type (WT) and the FFA-producing strain (dAS1T) of *S. elongatus* PCC 7942. PA is the major FFA species secreted by dAS1T and the concentration of PA tested in Fig. 1 was 60% higher than the maximum extracellular FFA level attained by dAS1T cultures in 240 h via FFA secretion [16]. Growth of the WT cells was somewhat slower in the presence of PA (Fig. 1a), but the appearance of the culture was similar to that of the control culture grown without PA (Fig. 1b). By contrast, growth of dAS1T was severely inhibited in the presence of PA. Cell growth ceased in 48 h after the inoculation (Fig. 1a) and the cultures turned blue in 96 h of cultivation (Fig. 1b). The inhibitory effect of exogenously added PA on the growth of dAS1T was to be ascribed to the reduced secretion of PA out of the cell. The dAS1T cells have a large capacity of FFA production but accumulate FFA to a toxic level once the rate of FFA production exceeds the rate of FFA excretion [16]. Although the SD277 mutant of *Synechocystis* sp. PCC 6803 was reported to attain an extracellular FFA concentration of 0.20 g L^−1^ [8], the present results indicated that this high external FFA concentration cannot be attained by dAS1T under the given conditions. The results also made it clear that removal of FFA from the medium is required to have the dAS1T cells excrete 0.13 g or more FFA per L of culture.

### Aqueous-organic two-phase cultivation with isopropyl myristate alleviated the toxic effects of FFA production on cell growth

**Fig. 2:**
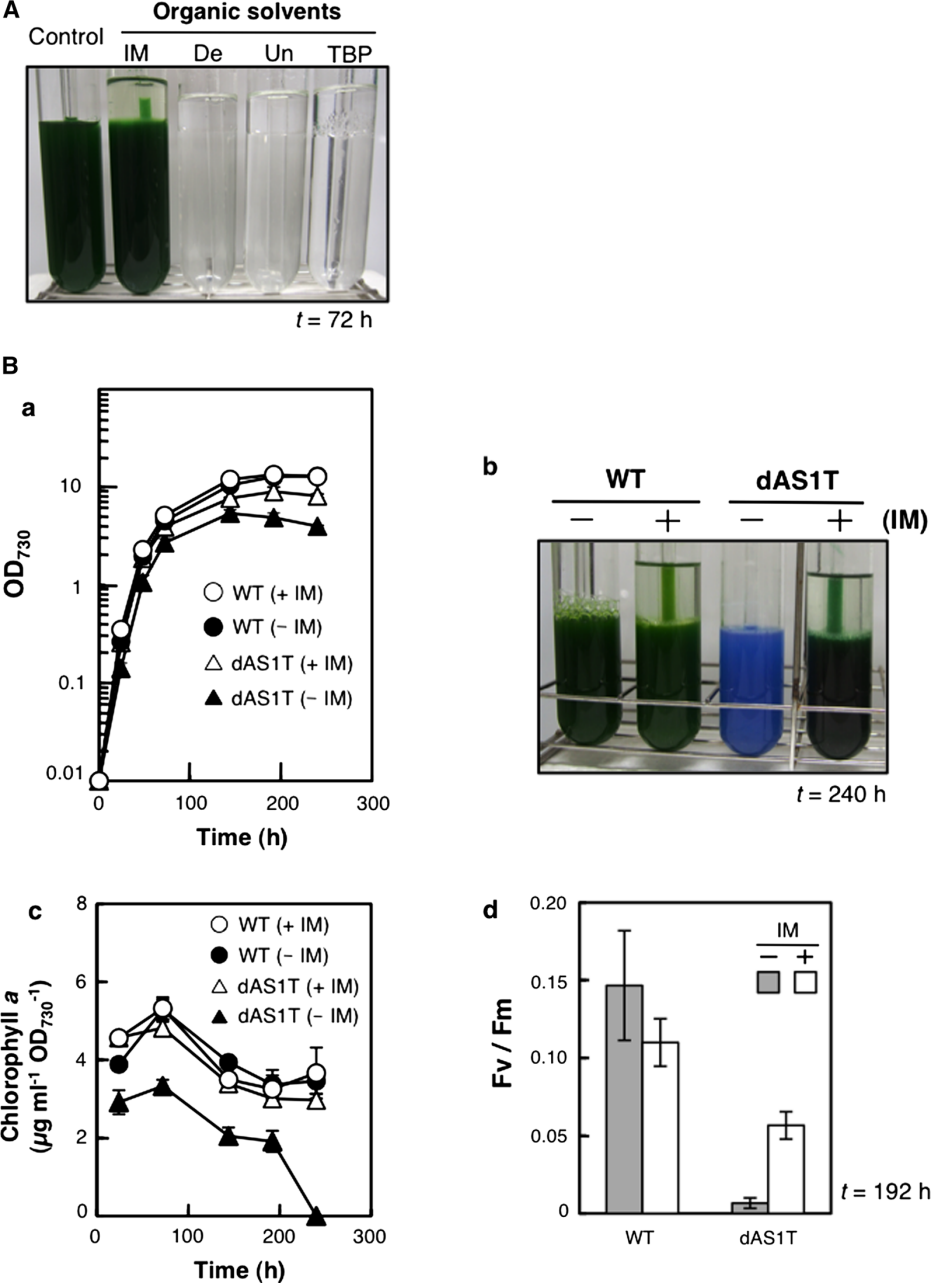
Development of an aqueous-organic two-phase culture system. **A** Effects of various hydrophobic solvents on the growth of *S. elongatus*. WT cells were inoculated into fresh medium to give an OD_730_ value of 0.01 and cultivated for 72 h with 10 mL each of the organic solvents indicated. *IM* isopropyl myristate, *De* decane, *Un* undecane, *TBP* tributyl phosphate. **B** Effects of the two-phase culture system with IM on growth (*a*, *b*), cellular chlorophyll content (*c*), and the photosynthetic yield (*d*). WT and dAS1T cells were grown either with or without 20 mL of IM for 240 h. Data shown in *a*, *c*, and *d* are the mean ± SE from biological triplicates. *Circles* WT; *triangles* dAS1T. *Open symbols* with IM; *filled symbols* without IM. *b* shows the appearance of the cultures at *t* = 240 h

Aqueous-organic two-phase culture system has been successfully used to enhance the production of hydrophobic compounds using engineered *E. coli* and *Saccharomyces cerevisiae* strains [17–19]. The two-phase cultivation system allows for removal of the product from the culture medium and thereby alleviates the toxic effects of the compound [20]. In an attempt to develop a two-phase culture system for the FFA production system using dAS1T, effects of decane (De), undecane (Un), tributyl phosphate (TBP), and isopropyl myristate (IM) on the growth of *S. elongatus* PCC 7942 were examined. Growth of WT cells was completely inhibited when the culture was overlaid with De, Un, or TBP, but not with IM (Fig. 2A). We hence used IM for further study.

Figure 2Ba, b compare the effects of IM on the growth of WT and dAS1T. The growth curves of the WT cells confirmed that IM had no adverse effects on cell growth (Fig. 2Ba). As previously observed [16], dAS1T cells grew slower than WT cells and died in 240 h of cultivation with complete loss of chlorophyll in the absence of IM (Fig. 2Ba, b). The cellular chlorophyll content of dAS1T was ~60% of the WT level during the first 200 h of cultivation and then sharply declined (Fig. 2Bc). When grown with IM, on the other hand, growth of dAS1T was very much improved and the cells remained green in 240 h (Fig. 2Ba, b), with the chlorophyll content being similar to the WT level during the entire course of cultivation (Fig. 2Bc). At *t* = 192 h, the photosynthetic yield of PSII, as determined by measuring the Fv/Fm ratio, was close to zero in dAS1T cultivated without the IM layer (Fig. 2Bd), indicating that PSII had been almost completely inactivated, whereas the cells cultivated with IM showed a significant Fv/Fm value, showing the operation of PSII (Fig. 2Bd). These results showed that the presence of the IM layer alleviated the adverse effects of FFA production in dAS1T.

### Two-phase culture system facilitated FFA secretion

**Fig. 3:**
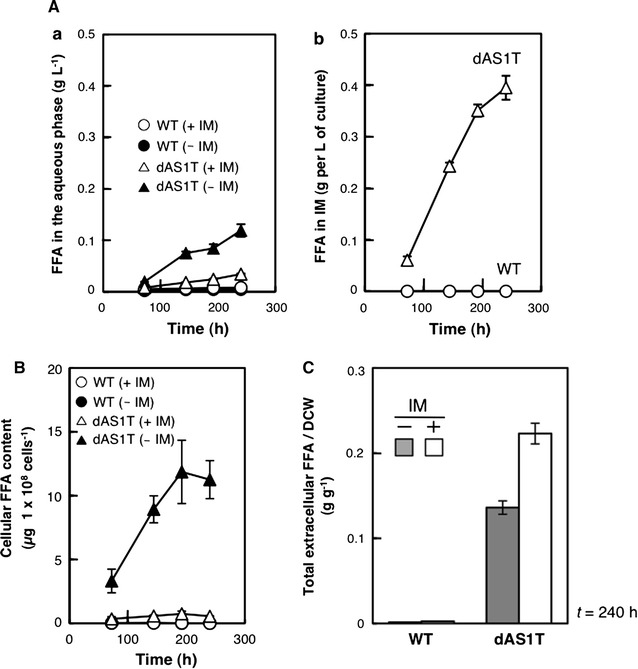
Effects of the two-phase culture system on FFA production. WT (*circles*) and dAS1T (*triangles*) cells were grown either with (+, *open symbols*) or without (−, *filled symbols*) IM. Data shown are the mean ± SE from the same biological triplicates as in Fig. 2B. **A** Time course of the accumulation of FFA in the aqueous phase (*a*) and the IM layer (*b*). The FFA concentration in IM was measured as described in the “Methods” section and converted into the amount of FFA produced per 1 L of culture by calculation. **B** Time course of cellular FFA content. **C** The yield of FFA per dry cell weight (DCW) at *t* = 240 h

Figure 3A shows the changes in the amounts of extracellular FFA during the experiment shown in Fig. 2B. For the cultures overlaid with IM, the amount of FFA in the IM layer as well as that in the aqueous layer was determined and expressed per unit volume of the culture (Fig. 3Ab). In the absence of IM, the dAS1T cells secreted 0.12 ± 0.01 g L^−1^ of culture in 240 h (Fig. 3Aa). In the presence of IM, only small amounts of FFA were found in the aqueous phase (0.034 ± 0.003 g L^−1^; Fig. 3Aa), but the IM layer accumulated large amounts of FFA, which reached 0.40 ± 0.02  g L^−1^ of culture at *t* = 240 h (Fig. 3Ab). Thus, the total amount of FFA secreted by dAS1T cells in the two-phase system was 3.6 times larger than that in the control culture grown without IM. In the two-phase system, more than 90% of the FFA secreted from the cells was found in IM, indicating effective extraction of FFA from the culture medium by the organic solvent. FFA in the IM layer was clearly not formed by degradation of IM, because no FFA was detected in the IM layer placed on the WT culture (Fig. 3Ab).

While the cellular FFA content of dAS1T increased sharply during cultivation in the absence of IM as previously observed (Fig. 3B), it remained low in the presence of the IM layer during the entire course of cultivation (Fig. 3B). These results indicated that extraction of FFA by IM from the culture medium facilitated secretion of FFA from the FFA-producing cells, allowing for robust growth of the cells up to *t* = 240 h.

Figure 3C shows the effects of IM on the yield of extracellular FFA per dry cell weight (DCW). In the presence of the IM layer, dAS1T excreted 0.22 ± 0.01 g of FFA per g of DCW in 240 h, which was 60% higher than the yield in the absence of IM (0.14 ± 0.01 g of FFA per g of DCW). Thus, the two-phase culture system increased the per-cell production of FFA.

### Two-phase culture system allowed for sustained production of FFA

**Fig. 4:**
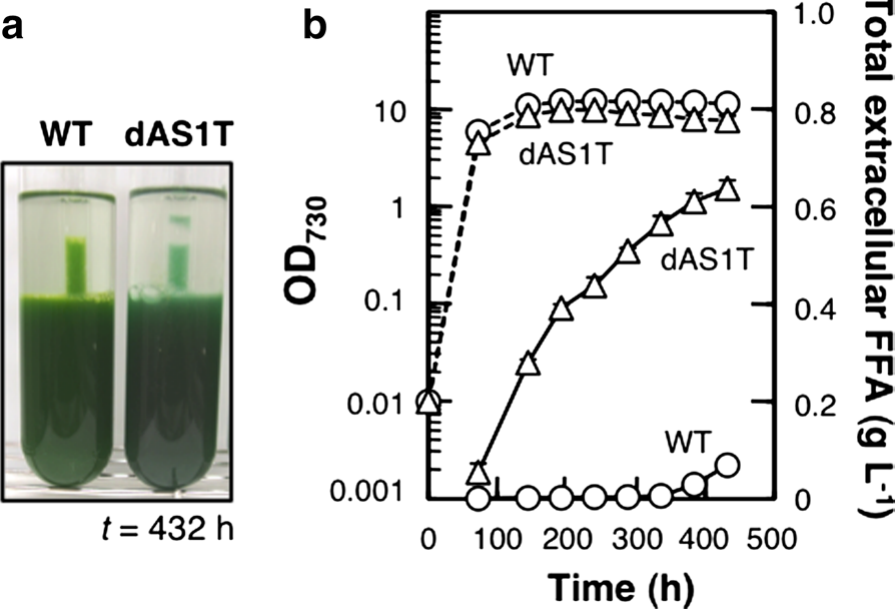
Long-term FFA production in the two-phase culture system. WT and dAS1T cells were grown with IM for 432 h. **a** Appearance of the cultures at *t* = 432 h. **b** Growth curves (*dashed lines*) and the changes of the total amount of extracellular FFA (i.e., the sum of FFA in aqueous and organic phases) (*solid lines*) during cultivation. *Circles* WT; *triangles* dAS1T. Data shown are the mean ± SE from biological triplicates

Given the stability of the dAS1T culture at *t* = 240 h in the presence of IM (Fig. 2), FFA production by dAS1T was investigated for a prolonged period of time (Fig. 4). In the presence of IM, dAS1T as well as WT survived up to *t* = 432 h without losing chlorophyll. The total amount of FFA secreted from the dAS1T cells reached 0.64 ± 0.02 g L^−1^ in 432 h. The highest rate of FFA excretion, which was obtained between *t* = 72 h and *t* = 144 h, was 3.1 ± 0.16 mg L^−1^ h^−1^ and the average rate of FFA excretion during the entire course of cultivation was 1.5 ± 0.04 mg L^−1^ h^−1^ (Fig. 4b). Since there was essentially no cell growth after *t* = 240 h (Fig. 4b), the per-cell yield of FFA was increased by the sustained FFA excretion to 0.36 ± 0.05 g g^−1^ of DCW after 432 h of cultivation with IM. These figures were 3–4 times larger than the highest records reported previously for the FFA-producing strains obtained from cyanobacteria [8, 9].

## Discussion

The FFA-producing strains constructed from *S. elongatus* PCC 7942 die out under high-light conditions due to intracellular accumulation of excess FFA, which results from the slower rate of FFA excretion as compared to the rate of FFA production in the cell [16]. We previously showed that FFAs can be secreted by both active transport and passive diffusion. *S. elongatus* PCC 7942 has two gene clusters encoding respectively a membrane component and a periplasmic component of a tripartite efflux pump of the resistance-nodulation-cell division (RND) family. Targeted disruption of one of these (*rndA1*–*rndB1*) rendered the cells highly sensitive to FFAs, indicating its involvement in FFA export [21]. Passive efflux of FFA was stimulated by removal of the hydrophilic O-antigen layer on the cell surface by gene manipulation [16]. However, the enhancement of passive efflux or that of the active FFA export could only partially rescue the growth defect of the FFA-producing strains [16, 21]. By contrast, the aqueous-organic two-phase culture system effectively enhanced FFA secretion and remarkably improved growth of the dAS1T strain (Figs. 2B, 3A, B). This is probably due to facilitation of passive diffusion of FFAs. The amount of FFA in the aqueous phase was decreased by the presence of the IM layer to 30% of that of the control culture, which seemed to have helped maintaining the FFA concentration gradient across the plasma membrane. These results indicated that removal of FFA from the culture medium is essential for the enhancement of FFA secretion and hence of FFA production.

The FFA productivity obtained with the dAS1T cultures overlaid with IM was the highest ever reported for cyanobacterial FFA-producing mutants. The cells excreted 0.64 g of FFA per L of culture in 432 h with an average FFA excretion rate of 1.5 mg L^−1^ h^−1^ (Fig. 4b). These figures are more than three times larger than those reported for the SD277 mutant of *Synechocystis* sp. PCC 6803, i.e., 0.20 g L^−1^ and 0.44 mg L^−1^ h^−1^ [8], and about five times larger than those reported for the S07 mutant of *Synechococcus* sp. PCC 7002, i.e., 0.13 g L^−1^ and 0.27 mg L^−1^ h^−1^ [9]. Unlike dAS1T, SD277 and S07 have some gene manipulations in addition to inactivation of *aas* and introduction of *‘tesA*. Increasing the activity of the CO_2_ fixation and fatty acid biosynthesis pathways were required in order to maximize FFA production [8, 9]. Since much higher FFA productivity was achieved in dAS1T simply by enhancement of FFA secretion, it is deduced that *S. elongatus* PCC 7942 naturally has a large capacity of CO_2_ fixation and fatty acid biosynthesis.

Ruffing previously reported that overexpression of ribulose-1, 5-bisphosphate carboxylase/oxygenase and acetyl-CoA carboxylase had adverse effects on the growth of *S. elongatus* FFA-producing mutants [22], effects of which can be accounted for by the toxic effect of enhanced accumulation of intracellular FFA. The observation also indicates that fatty acid biosynthesis activity of dAS1T can be further enhanced by these gene manipulations. To fully exploit the enhanced activity of fatty acid biosynthesis for FFA production, it would be required to enhance cellular activity of active FFA export. Even in the two-phase cultivation system, the color of the dAS1T cells but not the WT cells became slightly bluish at *t* = 432 h (Fig. 4a) and died in 450 h of cultivation (data not shown), suggesting intracellular accumulation of FFA after prolonged cultivation. Functional expression of FFA transporters is not simple in cyanobacteria [21], but is essential for further increase of the FFA productivity.

## Conclusions

FFA-producing cyanobacterial mutants secrete FFA, but accumulation of FFA in the medium interferes with the release of FFA from the cell. In the dAS1T mutant constructed from *S. elongatus* PCC 7942, intracellular FFA increases to a lethal level due to its large capacity of fatty acid biosynthesis. Removal of FFA from the medium facilitates FFA release from dAS1T cells and rescues them from FFA toxicity, enhancing the productivity of extracellular FFA to the highest level ever reported for cyanobacteria. Further increase of FFA production would require an enhancement of active FFA transport out of the cell.

## Methods

### Strains and growth conditions

A derivative of *S. elongatus* PCC 7942, which is cured of the resident small plasmid pUH24 (SPc), was used as WT in this study [23]. The FFA-producing strain designated as dAS1T was the *aas*-deficient mutant expressing a foreign thioesterase gene from *E. coli (‘tesA)* transcriptionally fused to the *nirA* promoter of *S. elongatus* PCC7942 [16]. The basal medium used in this study was a modification of the BG11 medium [24] prepared as described by Suzuki et al. [25]. The medium was buffered with 20 mM HEPES–KOH (pH 8.2) and supplemented with 15 mM KNO_3_ as a nitrogen source and when appropriate, supplemented with 0.1% ethanol and 0.13 g L^−1^ of palmitic acid (PA). WT and dAS1T cells were grown at 30 °C under high-light conditions (180 µE m^−2^ s^−1^) with aeration with 2% (vol/vol) CO_2_. In all the experiments, cells precultured under low-light conditions (50 µE m^−2^ s^−1^) to the exponential phase of growth (OD_730_ = 0.5–1.5) were used for inoculation of fresh medium, because the PSII activity of dAS1T sharply declines during the stationary phase of growth under the high-light conditions [16]. For two-phase cultivation of cyanobacterial cells, 50 mL of culture was overlaid with 20 mL of IM unless otherwise stated.

### Physiological measurement

Optical density at 730 nm (OD_730_) of the cultures was determined using a Shimadzu UV1700 spectrophotometer. Dry weight of cyanobacterial biomass in liquid cultures was estimated from OD_730_ of the cultures (Additional file 1: Figure S1). To determine the relationship between OD_730_ and dry cell weight (DCW) in a unit volume of culture, 10 mL aliquots of the cultures of various cell density (OD_730_ = 2.0–13.8) were vacuum-filtered through a pre-weighed MF-Millipore™ Membrane Filter (pore size: 0.45 µm, Merck Millipore). The cells on the filter were washed three times with 10 mL of deionized water and then dried at 75 °C until constant weight was reached. When DCW of the cultures overlaid with IM was measured, cells from 10 mL aliquots of the cultures were collected by centrifugation at 1700*g* for 10 min and washed three times with 10 mL of deionized water by resuspension and recentrifugation before vacuum filtration so that the effects of contaminating IM is minimized. For estimation of the amount of IM absorbed to the membrane filter, the BG-11 medium was overlaid with IM without cyanobacterial cells and incubated just as the cyanobacterial cultures. The IM-treated medium was passed through a pre-weighted membrane filter, and the filter was washed and dried for weighing. Twenty-six sets of data obtained with independent cultures were fitted to a linear relationship (*y* = 0.255*x* − 0.144, *r* ^*2*^ = 0.942) (Additional file 1: Figure S1), which was used for estimation of the cell dry matter content of a culture from the OD_730_ value.

Chlorophyll was determined as described by Mackinney [26] and the content was normalized to OD_730_. The photosynthetic yield of PSII was determined using an AquaPen-C fluorometer (AP-C100, Photon Systems Instruments).

### FFA analysis

The concentration of FFA in the culture medium (or the aqueous layer in the two-phase cultivation system) and the cellular FFA content were determined as described by Kato et al. [16]. The concentration of FFA in the IM layer was enzymatically determined using the Free Fatty Acid Quantification Kit (BioVision) as follows. 1 μL aliquots of the IM layer was mixed with 49 μL of Assay Buffer in the kit in a 96-well plate and then analyzed according to the manufacturer’s instruction. The volume of the IM layer used for FFA determination was limited to one-fiftieth of the assay mixture, because larger amounts of IM caused artifactual coloration at 570 nm in the absence of FFA (Additional file 2: Figure S2). Absorbance at 570 nm was measured using a Multiskan™ GO microplate spectrophotometer (Thermo Fisher Scientific). A standard curve for FFA was generated for each measurement, using a concentration series of PA dissolved in IM (0, 2, 4, 6, 8, 10 mM) (Additional file 3: Figure S3). The amount of FFA in the IM layer was normalized to the culture volume and expressed in g L^−1^.

## Additional files

**Additional file 1: Figure S1.** The relationship between OD_730_ and the dry cell matter content of the cyanobacterial cultures. Dry cell weight (DCW) per L of cyanobacterial cultures (*Y*-axis) and optical density at 730 nm (OD_730_) of the cultures (*X*-axis) were determined as described in “Methods” section. Data from 26 cultures were fitted to a linear relationship: *y* = 0.255*x* − 0.144 (*r* ^*2*^ = 0.942). Data shown are from 26 samples of *S. elongatus* PCC7942 cultures.

**Additional file 2: Figure S2.** Effects of IM on determination of FFA using the Free Fatty Acid Quantification Kit (BioVision). Fresh IM (circles) and the IM layer incubated for 240 h with the culture of dAS1T (triangles) were mixed with Assay Buffer in the kit at various ratios to give a total volume of 50 µL and then analyzed according to the manufacturer’s instruction.

**Additional file 3: Figure S3.** A standard curve for FFAs dissolved in isopropyl myristate. A concentration series of palmitic acid (PA) dissolved in isopropyl myristate (IM) (0, 2, 4, 6, 8, 10 mM) was analyzed using the Free Fatty Acid Quantification Kit (BioVision) as described in the text. One of the essentially same results obtained in more than ten independent measurements is shown.

## Abbreviations

Aas: acyl-ACP synthetase
ACP: acyl carrier protein
Chl: chlorophyll
De: decane
DCW: dry cell weight
FFAs: free fatty acids
IM: isopropyl myristate
PA: palmitic acid
RND: resistance-nodulation-cell division
SPc: small plasmid cured
TAG: triacylglycerol
TBP: tributyl phosphate
Un: undecane
WT: wild type
