# Supplementary material for: Removal of the product from the culture medium strongly enhances free fatty acid production by genetically engineered Synechococcus elongatus
Source: Biotechnol Biofuels. 2017 May 31;10:141. doi: 10.1186/s13068-017-0831-z (PMC5452621; doi:10.1186/s13068-017-0831-z)
Supplement: Supplementary file 1 — Additional file 1: Figure S1. The relationship between OD730 and the dry cell matter content of the cyanobacterial cultures. Dry cell weight (DCW) per L of cyanobacterial cultures (Y-axis) and optical density at 730 nm (OD730) of the cultures (X-axis) were determined as described in “Methods” section. Data from 26 cultures were fitted to a linear relationship: y = 0.255x − 0.144 (r 2 = 0.942). Data shown are from 26 samples of S. elongatus PCC7942 cultures. [file 13068_2017_831_MOESM1_ESM.pdf]

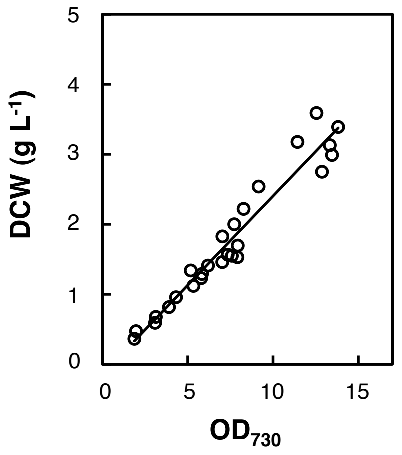

Fig S1. The relationship between OD_{730} and the dry cell matter content of the cyanobacterial cultures

Dry cell weight (DCW) per L of cyanobacterial cultures (*Y*-axis) and optical density at 730 nm (OD_{730}) of the cultures (*X*-axis) were determined as described in Methods section. Data from twenty-six cultures were fitted to a linear relationship: y = 0.255x - 0.144 (r^2 = 0.942). Data shown are from twenty-six samples of *S. elongatus* PCC7942 cultures.
