# Supplementary material for: Removal of the product from the culture medium strongly enhances free fatty acid production by genetically engineered Synechococcus elongatus
Source: Biotechnol Biofuels. 2017 May 31;10:141. doi: 10.1186/s13068-017-0831-z (PMC5452621; doi:10.1186/s13068-017-0831-z)
Supplement: Supplementary file 2 — Additional file 2: Figure S2. Effects of IM on determination of FFA using the Free Fatty Acid Quantification Kit (BioVision). Fresh IM (circles) and the IM layer incubated for 240 h with the culture of dAS1T (triangles) were mixed with Assay Buffer in the kit at various ratios to give a total volume of 50 µL and then analyzed according to the manufacturer’s instruction. [file 13068_2017_831_MOESM2_ESM.pdf]

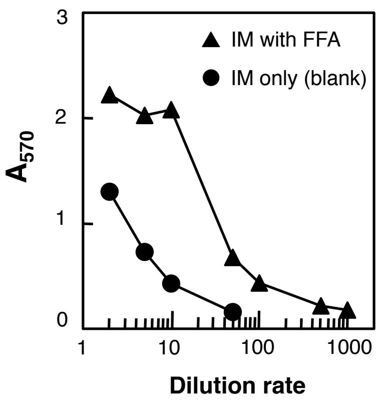

Fig S2. Effects of IM on determination of FFA using the Free Fatty Acid Quantification Kit (BioVision).

Fresh IM (circles) and the IM layer incubated for 240 h with the culture of dAS1T (triangles) were mixed with Assay Buffer in the kit at various ratios to give a total volume of 50 μL and then analyzed according to the manufacture's instruction.
