# Supplementary material for: Removal of the product from the culture medium strongly enhances free fatty acid production by genetically engineered Synechococcus elongatus
Source: Biotechnol Biofuels. 2017 May 31;10:141. doi: 10.1186/s13068-017-0831-z (PMC5452621; doi:10.1186/s13068-017-0831-z)
Supplement: Supplementary file 3 — Additional file 3: Figure S3. A standard curve for FFAs dissolved in isopropyl myristate. A concentration series of palmitic acid (PA) dissolved in isopropyl myristate (IM) (0, 2, 4, 6, 8, 10 mM) was analyzed using the Free Fatty Acid Quantification Kit (BioVision) as described in the text. One of the essentially same results obtained in more than ten independent measurements is shown. [file 13068_2017_831_MOESM3_ESM.pdf]

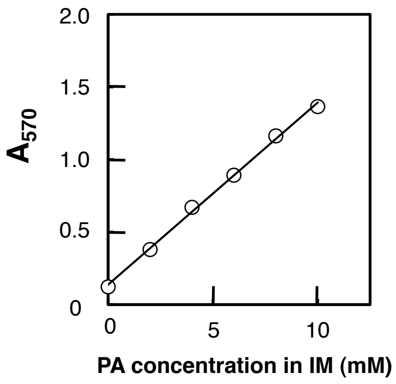

Fig S3. A standard curve for FFAs dissolved in isopropyl myristate

A concentration series of palmitic acid (PA) dissolved in isopropyl myristate (IM) (0, 2, 4, 6, 8, 10 mM) was analyzed using the Free Fatty Acid Quantification Kit (BioVision) as described in the text. One of the essentially the same results obtained in more than 10 independent measurements is shown.
